# Effects of School-Based Neurofeedback Training on Attention in Students with Autism and Intellectual Disabilities

**DOI:** 10.1007/s10803-024-06400-8

**Published:** 2024-05-28

**Authors:** Michał Gacek, Tomasz Smoleń, Łukasz Krzywoszański, Agnieszka Bartecka-Śmietana, Beata Kulasek-Filip, Maja Piotrowska, Dominika Sepielak, Katarzyna Supernak

**Affiliations:** 1 Institute of Psychology, Jagiellonian University, ul. Ingardena 6, 30-060 Krakow, Poland; 2 Department of Cognitive Science, Jagiellonian University, ul. Grodzka 52, 31-044 Krakow, Poland; 3 Institute of Psychology, The Pedagogical University of Krakow, ul. Podchorazych 2, 30-084 Krakow, Poland; 4 Special Education and Child Care Center No. 1 in Krakow, ul. Barska 45, 30-307 Krakow, Poland

**Keywords:** Autism, Intellectual disability, Neurofeedback, Special education, Sustained attention, Selective attention

## Abstract

In this study we aimed to assess the influence of school-based neurofeedback training on the attention of students with autism and intellectual disabilities. We assessed 24 students of a special education center who attended neurofeedback training sessions during the schoolyear; we also assessed 25 controls from the same center. We used two computer tasks to assess sustained attention in simple and cognitively demanding test situations, and we used a pen-and-paper task to assess selective attention. Each student who took part in the study was tested at the beginning and at the end of the schoolyear. Students from the experimental group significantly improved their performance in the task related to sustained attention to simple stimuli. No performance improvement related to neurofeedback treatment was observed in either sustained attention in cognitively demanding situations or selective attention. School-based neurofeedback training may improve sustained attention to simple stimuli in students with developmental disabilities.

## Introduction

Neurofeedback (NFB) is an increasingly popular form of treatment that is used in a wide range of therapeutic applications (Dessy et al., 2018). During NFB training, participants learn how to regulate bioelectrical brain activity by recognizing patterns in their responses and developing strategies which help them to obtain self-control over their brain functions (Hammond, 2006). During NFB sessions, participants are usually involved in game-like activities in which they need to control objects presented on the screen by adjusting their brain-wave patterns to parameters required by therapists. Improvements in regard to desired brain-wave patterns produce effects on the screen that are visible to participants, thus reinforcing their responses and making it possible to activate these responses outside of the training conditions (Marzbani et al., 2016).

Studies on the efficacy of NFB treatment indicate positive effects of this type of training in enhancing the performance of participants from the general population, as well as in improving the functioning of various clinical groups. In participants from the general population, NFB training has been reported to improve professional performance in groups such as surgeons, musicians, and athletes. In the clinical context, NFB has been reported to alleviate symptoms of various conditions, including epilepsy, depression, anxiety, PTSD, ADHD, and others (for a review see Niv, 2013 or Marzbani et al., 2016). The efficacy of NFB training was tested in regard to its effects on real-life situations, indicating that this type of training may contribute to changes in behavior, such as reduction of cigarette cravings in heavy smokers (Kim et al., 2015), increase in control over anxiety in a group with high contamination-related anxiety (Scheinost et al., 2013), or improvement in symptoms in patients with obsessive–compulsive disorder and Tourette syndrome (Rance et al., 2018). Although NFB requires special electronic equipment to record and analyse electrical brain activity, including electrodes that measure brain activity, the method itself is not invasive and may be considered a reasonable alternative to other forms of therapy, such as psychopharmacology (Coben, 2013; Medici et al., 2018). However, studies regarding NFB training are also criticized for their frequent dependence on subjective indicators of effectiveness, such as self-report measures or third-party assessment, and their lack of employment of rigorous methodological criteria (Schabus et al., 2017; Thibault & Raz, 2016; Thibault et al., 2017).

Attempts to improve attentional functioning through NFB training go back to the 1970s (Lubar & Shouse, 1976; Shouse & Lubar, 1979). Meta-analyses of the efficacy of NFB in participants diagnosed with ADHD have reported positive changes in inattention symptoms after training, and these changes were greater than those regarding hyperactivity and impulsivity (Arns et al., 2009; Cortese et al., 2016; Micoulaud-Franchi et al., 2014; Van Doren et al., 2018). Typically, the efficacy of NFB training in participants with ADHD is assessed by third-party ratings (Arns et al., 2020). The positive effect of NFB training on inattention in this group is strongly supported in studies in which training efficacy was estimated by most-proximal evaluators, usually parents of participants (Riesco-Matias et al., 2021). Another group in which improvement in attentional functioning was observed after NFB training was children with learning disabilities, whose performance increased in experimental tasks more than that of controls (Fernandez et al., 2007). Positive effects of NFB on attention were also found in healthy participants from the general population (see Angelakis et al., 2007). However, studies in which different kinds of behavioral measures of cognitive functioning were used report mixed results (Pamplona et al., 2020; Wang & Hsieh, 2013), thus suggesting that improvement in attentional functioning after NFB training may involve only certain aspects of attention, mainly those related to sustained attention.

Studies on the efficacy of NFB training in participants with autism and intellectual disabilities report positive effects of such training on people in these groups (Niv, 2013). Several studies involving autistic individuals in which parents assessed treatment efficacy by completing the Autism Treatment Evaluation Checklist (ATEC) reported a decrease in autism symptoms after NFB training (Coben & Padolsky, 2007; Jarusiewicz, 2002; Pineda et al., 2008). In a study by Kouijzer et al. (2009a), parents reported improvements in social interaction, communication and attenuation of typical autistic behavior following NFB treatment. Consistently with previous results, a study by Friedrich et al. (2015) showed that parental reports after NFB training also indicated improvements in autistic individuals in ATEC. In this study, parental assessment also indicated improvement in adaptive behavior and social responsiveness.

Although parental reports seem to confirm the efficacy of NFB training in the functioning of autistic individuals, the effects of such training on attention in studies in which objective test measures were employed are not conclusive. Since atypical attentional functioning is a common characteristic of autism (see Ames & Fletcher-Watson, 2010), generalizations should not be made hastily regarding the results of NFB training obtained in other groups, such as children with ADHD. Kouijzer et al., (2009a, 2009b) tested cognitive abilities of several children with autism in relation to NFB training and reported an increase in selective attention. These authors found that effects of training were maintained 12 months later in the follow-up study. Improvements in selective attention were also reported in a study by Saleem and Habib (2023), but this study did not have a control group, therefore the results are difficult to interpret. The results of another study by Kouijzer et al. (2010) indicated that NFB training did not influence attentional control or most executive functions in participants with autism. Interestingly, in that study the parental reports indicated improvements in participants’ functioning after NFB training, but the teachers’ reports did not. In a study by Pineda et al. (2008), high-functioning autistic individuals obtained increased scores after NFB training in a sustained attention task. Also, Mercado et al. (2021) reported improvements in regard to various aspects of attentional functioning in autistic participants after NFB training.

Studies of the effects of NFB training on attention in people with intellectual disability are scarce and, to the best of our knowledge, have been conducted only on small samples of up to 10 participants in experimental groups. Breteler et al. (2012) reported positive post-training changes in attention in a group of residential children with intellectual disability. In another study, positive changes regarding ADHD symptoms after NFB training were observed in several children with Down syndrome (Surmeli & Ertem, 2007), but this study did not include a control group. Positive effects of NFB training on attention have been reported in a group of children with intellectual disability who were compared to children subjected to visual perception training and to controls who did not participate in any type of training (Hong & Lee, 2012).

## Aim of the Study

In this study, we aimed to assess whether school-based neurofeedback training improves the attention of students with autism and intellectual disabilities. Testing the efficacy of school-based NFB training is important because special education centers in Poland can apply for public funds to buy NFB equipment and cover the costs of training and therapists. However, whether the costs of implementing NFB therapy are proportionate to its effects remains unknown. A review of NFB therapy in autism spectrum disorder by Coben et al. (2010) referred to NFB therapy as “possibly efficacious” and having the potential to improve functioning within this population. Another review by Holtmann et al. (2011) rather discouragingly concluded that the current evidence is not sufficient to support NFB as treatment for autism spectrum disorder and that it is unknown whether this type of therapy is more effective than existing interventions. This negative appraisal of NFB concerns the high costs of implementation and the limited data regarding NFB’s efficacy in regard to autistic individuals, especially in comparison to other forms of treatment. On the other hand, Coben (2013) presents a more positive view on the matter, stating that NFB treatment has the potential to provide long-term improved functioning of people with autism that is related to changes in brain activity, and that this treatment may be preferable to other forms of therapy, such as drug interventions, due to the lower risk of negative side effects. Thus, according to this author, NFB training should be provided to people with autism spectrum disorder, and its effects should be studied further.

We decided to test the effects of NFB on attention since several studies have shown that this type of training may positively influence attentional functioning in various clinical and non-clinical groups (e.g., Dessy et al., 2018; Niv, 2013; Riesco-Matias et al., 2021). However, attention is a complex topic for which there are various definitions that are usually related to specific experimental paradigms (see Styles, 2006 or Lindsay, 2020). In our study, we chose to assess two simple types of attention, i.e., selective attention and sustained attention, and one compound aspect of attention, which we labeled as sustained attention in cognitively demanding situations. Both selective and sustained attention are considered important in regard to education (for a review, see Gallen et al., 2023 and Stevens & Bavelier, 2012). Selective attention requires searching the perceptional field and selecting one type of stimuli while ignoring others; this type of attention may be important in education as it is involved in reading and mathematics. Sustained attention is related to concentration on tasks for a prolonged time, which is necessary for basic school activities, such as following instructions and lectures. In our study, we also assessed sustained attention in cognitively demanding situations which required inference and focus on information based on the obtained feedback.

We decided to use objective test measures to assess attention because, in our opinion, they are more credible indicators of changes related to NFB treatment than third-party reports. We developed tasks based on descriptions of measures used in experimental cognitive psychology studies. The first task, which assessed selective attention, was a paper–pencil version of the family of tasks from visual search paradigms (Treisman, 1977; Treisman & Gelade, 1980). Visual search related to selective attention is often studied in regard to autism, and some studies indicate that autistic individuals may achieve better results in this area than controls (e.g., Joseph et al., 2009; Shirama et al., 2016). However, considering the fact that our group included people with intellectual disability, who typically experience significant limitations in regard to attentional functioning (Hronis et al., 2017), we decided to simplify our task by using the only feature of the stimuli that was important in making the choice (shape), and we made the difference between signal and noise subtle enough to require serial scanning of the stimuli. Also, in this task we used a much larger set of stimuli than those typically used in other versions of visual search tasks. The second task, which assessed sustained attention, was a computer version of the Mackworth (1948) Clock Task. The sole modification was a reduction of the overall task duration to adapt the task to the capabilities of our participants. Similar tasks to measure sustained attention have been employed in other studies on autistic individuals (Alloway & Lepere, 2021; Johnson et al., 2007). The third task we used tested sustained attention in a cognitively complex situation which required inference and focus in order to find a correct response and use it consequently. This task was a slightly modified version of the one used in a study by Gacek et al. (2017) to assess cognitive performance of students with mild intellectual disability.

In our study, NFB training was provided at a public special school at no cost to the participants’ parents. Due to the fact that there are relatively few studies on the efficacy of NFB in people with autism and intellectual disability, we assumed that attentional abilities should be particularly susceptible to enhancement in the course of NFB training. Providing data on the outcomes of NFB training could serve as an argument in the discussion regarding further popularization of NFB in special schools that justifies the expenses related to the implementation of this method in public institutions. The research hypothesis in our study included an interaction effect of test (pretest/posttest) and group (trained/untrained) on all dependent variables, i.e., selective attention, sustained attention to simple stimuli, and sustained attention in a cognitively demanding task. In the post-training assessment in the group that went through the NFB training, we assumed that the performance in each of the three attention tasks would be higher than in the pre-training assessment when compared to the group that did not undergo the NFB training.

## Method

### Ethical Considerations

We obtained the approval of the ethical committee at Jagiellonian University (approval number KE/50_2021) and the school board of the center where we conducted the research. Informed consent was obtained from all participants and their parents. The data was kept and used in accordance with the General Data Protection and Regulation Law in force (European Parliament, 2016).

### Participants

We assessed 49 students of a special education center in Poland. The center where the study was conducted accepts only students from 16 to 24 years old with diagnoses of intellectual disability or autism. These students take part in either a specialized vocational preparation training program for students with autism and mild intellectual disability, or in a general vocational preparation program for students with moderate or severe intellectual disability and combined disabilities. Each program takes three years to complete; this period may be prolonged at the request of legal guardians, dependent on the opinion of teachers and school psychologists. The students are accepted to the center based on diagnoses provided by psychological-pedagogical counselling centers, where students are assessed by teams of specialists, including doctors, pedagogists, and psychologists. The diagnostic assessment of students who participated in the study was made during their final year of primary school, several months before applying for acceptance by the special education center. The students’ diagnoses were given in accordance with ICD-10 criteria (World Health Organization, 2004).

The study was not preceded by a power analysis for two main reasons. Firstly, our intention was to include as many subjects as feasible, given the prevailing circumstances. As such, the anticipated number of subjects as determined by a power analysis would not have been a practical benchmark. Secondly, due to limited data regarding the effects of NFB on various aspects of attention in the groups that participated in the study, estimating the effect size was prone to considerable uncertainty, potentially biasing the estimated number of participants and introducing errors. The experimental group comprised 24 students (18 boys, *M*_*age*_ = 18.08; *SD* = 1.38) who attended school-based neurofeedback training during the schoolyear, and 25 controls (20 boys, *M*_*age*_ = 17.3; *SD* = 1.74) who had a similar school curriculum as the other group but did not participate in neurofeedback training. The students’ parents were informed about the possibility of participation in neurofeedback training at the beginning of the school year. The treatment was provided once a week for the whole school year, at no additional cost to the participants’ parents. Prior to the training, all participants were required to obtain approval from a neurologist stating that there are no contraindications for participation in NFB therapy. The standard procedure regarding the neurologist’s approval required analysis of EEG results, interviews with the student and parents, and a review of the medical history of the participant. In the experimental group in our study, we tested only students who had just begun the NFB training at the center. The controls either did not apply to attend the NFB training or they did not provide approval from a neurologist to attend the training.

In the experimental group, eight students were diagnosed with autism, 14 with intellectual disability, and two were diagnosed with autism combined with intellectual disability. In this group, five students with intellectual disability also had diagnoses of mild hearing loss or physical disability. In the control group, seven students were diagnosed with autism, 16 with intellectual disability, and two with autism combined with intellectual disability. In this group, two students with intellectual disability also had diagnoses of physical disability. In each case, the diagnoses were provided by a psychological-pedagogical counselling center. Participants with physical disabilities did not have difficulties that would hinder their functioning in manual tasks, such as pressing the keys on the laptop or using a pen. These participants did not require additional adjustments regarding their condition. Participants with hearing loss were provided with hearing aids, and they did not show verbal communication deficits. They were also able to react properly to the sound in the Avoidance Learning Task, as indicated by their performance in the training sequence in this task.

### Measures

In this study we utilized four tasks, two of which (Raven’s Progressive Matrices and Mackworth Clock Test) have a long history of usage and are firmly established in the field. The remaining two tasks (Flags and Stars Task, and Avoidance Learning Task) were specifically designed for the purposes of this study. Considering that our study involved participants who might differ significantly as regards their intellectual functioning and that studies into the effects of NFB training on people with intellectual disability are scarce and provide limited data, the RPM score was used to control an important co-variate, namely intelligence, which could potentially explain a meaningful portion of the model’s variance. In a pilot study conducted a year prior to the actual study, we assessed four students with diagnoses of autism and intellectual disability who attended the center at which we planned to conduct the research. During the pilot study, we tested whether the tasks we developed were not too frustrating for participants and could be used with a low risk of obtaining floor or ceiling effects. Based on the obtained results, we detailed the procedures for administering all tasks and adhered to these protocols throughout the study.

*Raven’s Progressive Matrices* (RPM) is a measure developed primarily to assess eductive, or “meaning-making”, ability (Raven et al., 2000). This measure is considered a prominent indicator of general fluid intelligence (e.g. Carpenter et al., 1990; Ren et al., 2014). In our study, we used the standard version of RPM, which consists of 60 matrices that differ in difficulty.

**Fig. 1:**
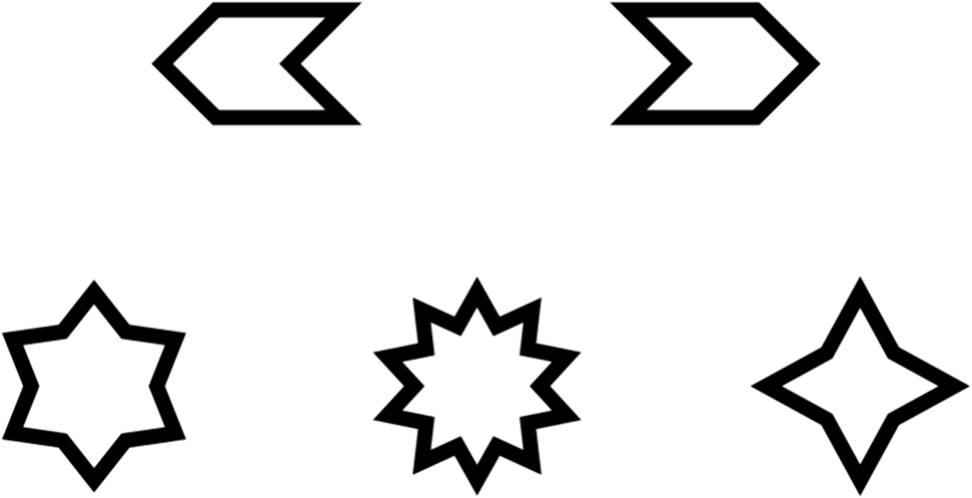
Stimuli presented in the Flags and Stars Task. Upper row: flags—signal (on the left) and noise. Bottom row: stars—signal (on the left) and noise

*Flags and Stars Task* (FST) is the measure we used to assess selective attention. We based this task on the Clock Test measure of attention, in which a person is presented with a series of 400 stimuli depicting small clocks. Most of these clocks show a certain time, for example 4 o’clock, while 10% of the clocks show a different time, for example 6 o’clock. In this task, the participant scans the clocks in order, marking those that show a different time than the target time (for details, see Towey et al., 2019). Our version of this task comprised two parts. In the first part, participants were presented with three sheets of paper depicting a total 1767 flags in 93 rows of 19 flags (approximately 8 × 5 mm, see Fig. 1, upper row). The participants scanned the flags in the given order (from left to right, row by row) and marked the target stimuli (flags pointing left). Among the 1767 stimuli, there were 88 (5%) target stimuli and 1,679 distractor/noise stimuli (flags pointing right).

In the second part of the task, participants were presented with sheets of paper depicting stars with four, six, or ten points (see Fig. 1, bottom row). Stars were arranged in 66 rows of 19 stars (total of 1254). Among them were 64 (5%) six-point target stars and 1190 four- or ten-point distractor/noise stars in equal proportions. Similarly as in the first part, participants were instructed to scan the stars in order and mark the targets. Each part of the task was preceded with a short training session during which the experimenter assessed whether the participant understood the instruction and was able to complete the task. Each part of the task lasted for 2 min, after which the experimenter marked the place on the sheet of paper where the participant had finished.

*Avoidance Learning Task* (ALT) is the measure we used to assess sustained attention in cognitively demanding situations. Similar measures are often employed to assess learning effectiveness in learned helplessness paradigm studies (see, for example, Sedek & Kofta, 1990. or Gacek et al., 2017). In this type of task, participants are presented with stimuli which are associated with certain keys on the keyboard. They need to press the correct key associated with a stimuli to avoid an unpleasant sound coming from the speakers. Our version of this task comprised three trials, each of which comprised a sequence of ten repeats of the stimuli. In each trial, the participant was presented with color squares on the screen and was instructed to try and associate the squares with the marked keys on the keyboard in order to prevent the unpleasant sound. We marked the letters Z, X, and C on the keyboard with yellow, blue, and red paper and told the participants that these were the active keys in this task. In each trial, the correct keys were randomly selected. In the first trial, one of the keys prevented the sound if it was pressed while the square associated with the stimuli was presented on the screen. In the second trial, participants again needed to find the correct key, but this time two squares of different colors were presented in sequence. Pressing only one key associated with one of the squares was necessary to prevent the sound. In the third trial, two keys on the keyboard were associated with two differently colored squares. The participant’s reaction in this task was considered correct each time in each trial they pressed a key (or a sequence of keys) that they had not pressed before while trying to find the correct way to prevent the sound; after the participant had found the key which stopped the sound for the first time in a trial, each subsequent prevention of the sound was counted as a correct response. We assumed that such accuracy measure scoring should be related to sustained attention towards performance in a cognitively demanding situation which requires finding and repeating the correct answer. The task was preceded by a short training sequence.

**Fig. 2:**
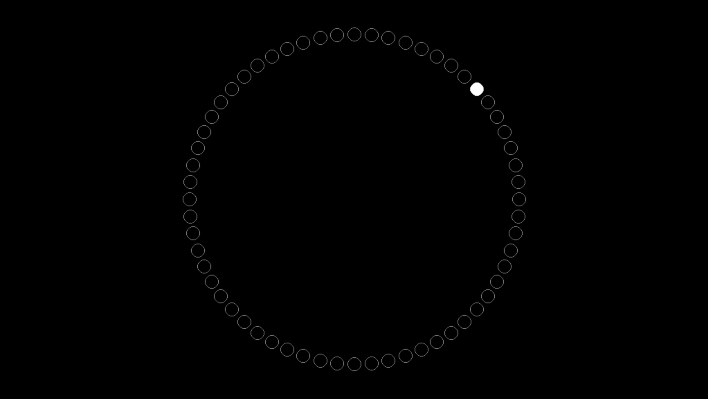
Example screen of the Mackworth Clock Test

*Mackworth Clock Test* (MCT) is a measure that assesses sustained attention to simple stimuli; it was developed by Mackworth (1948) and has subsequently been used in various versions and samples (see Lichstein et al., 2000). We used the computer-based version of this measure, in which participants watched a simplified clock consisting of a circle of sixty points on the screen (Fig. 2). One of the points is marked with a white dot. Every 1.5 s, the mark moved to the next point on the clock. At random intervals, the dot skipped one point and moved after not 1.5 but 3 s. Participants were instructed to react to each such ‘double jumps’ by pressing the space key. On average, the double jumps occurred every 31 s. The task consisted of 952 noise trials and 48 target trials, resulting in a 25-min-long assignment. The task was preceded with a training session during which participants were asked to react to three double jumps on the screen. The training session lasted 1.5 min and participants were informed that it would include exactly three double jumps. Detailed feedback on participants’ performance was given after the training.

### Procedure

Each student who took part in the study was tested with three attentional tasks at the beginning and the end of the schoolyear. The NFB training was provided by two therapists who had completed a two-level NFB training certification program based on the curriculum developed by the Biofeedback Certification Institute of America; before they had started to provide NFB therapy, both the therapists had observed this therapy in practice and had been supervised for at least a year by an experienced neurofeedback practitioner. Students in the experimental group participated in the NFB training, which consisted of 13 to 29 weekly sessions over a total duration of 35 weeks, with breaks for school holidays and student absences. Each training session lasted 45 min, with the NFB training itself lasting approximately 25–30 min. The session consisted of several rounds of 4 to 6 min each, separated by breaks of approximately 1 min. After each completed round, a scoreboard in the form of a bar graph showing the number of points scored was displayed on the screen. During each session, the therapists commented on the participants’ performance and encouraged them to continue the training. The therapists gave positive verbal feedback when the participants’ performance indicators increased. When participants could not obtain the desired outcome, the therapists explained that this was due to external causes, such as being tired that day, and that the participant should be able to make progress. The therapists established what they would say and when at the beginning of the school year as part of the standard procedure.

The training sessions took place during school hours, between 8 a.m. and 3 p.m. at the special education center. The office where the training took place was located in a part of the building away from the classrooms, therefore the noise made by other students during school activities was not heard. The office was equipped with a chair with a footrest for the participants, and a 32-inch TV was set up in front of it for presentation of the training feedback. The therapist used a computer desk to allow eye contact when interacting with the participants.

An Ag/Cl cup electrode mounted on TEN20 adhesive electroconductive paste was used as the recording electrode for the EEG signal. Two ear clip electrodes attached to the auricular flap served as neutral and reference electrodes. The skin at the site of electrode application was pre-cleaned with saline solution. The EEG signal was amplified and recorded at a sampling rate of 250 Hz using the unipolar channel of a two-channel DigiTrack BF EEG head, manufactured by ELMIKO BIOSIGNALS Ltd. (Poland), equipped with a 24-bit analogue-to-digital converter. The impedance was kept below 20 kΩ and the electrode connection was adjusted if this was exceeded.

The SMR/theta protocol was used to train all students, with the recording electrode positioned at either the C_Z_ or C_4_ site. This type of training has been reported to have positive effects on cognitive performance, enhancing attention and working memory (Campos da Paz et al., 2018; Vernon et al., 2003). The guidelines for the training were based on the current literature (Enriquez-Geppert et al., 2017 and Micoulaud-Franchi et al., 2015). If low amplitude in the beta band was observed in the power spectrum of the recorded EEG, the beta/theta protocol was also included in the training, with the recording electrode positioned at the C_3_ site. The training threshold was controlled by the therapists and was adjusted in real time according to the current level of training performance. Feedback on the current performance in regard to the selected training threshold criteria was presented to the participants by temporarily activating a simple electronic game or animation displayed on the TV screen. The feedback display module used in a given session could be visual or audio-visual and was chosen by the students from over a dozen available options. Additional feedback and motivational reinforcement were provided by verbal messages from the therapist.

### Statistical Analysis

In the FST, the dependent variable was sensitivity index (*d*’)—a measure of performance in binary decision tasks which includes both omissions and false alarms in order to take into account the possible biased strategy of a participant (Stanislaw & Todorov, 1999). Summed performance in both the flags and stars parts was analyzed. In the ALT, the dependent variable was the compound accuracy measure, which is described in the Measures section. In the MCT, the dependent variable was the number of signal omissions. This variable was identical but opposite to the number of correct signal detections (hits) because these two summed up to the number of signals presented in each performance of the task (48). In the process of preparing the measures, we tested a small group of people with developmental disabilities who did not take part in the main study; we observed that the tested persons almost never committed false alarms, so we did not include them in the analysis.

In the analyses of the FST and ALT results, a linear mixed model with random effect of participant on intercept was used. The results of this model’s fit were provided to the ANOVA. Because the dependent variable in the MCT had the form of the number of successes, in a fixed number of trials a generalized mixed linear model with binomial distribution was used in the analysis. This mixed model included a random effect of participant on the intercept. The results of this model’s fits were provided to the analysis of deviance, which is a tool similar to ANOVA but based on examination of the deviance instead of the variance. The analysis of deviance was based on the χ^2^ distribution instead of the *F* distribution. This tool was used because it allowed us to obtain *p*-values for the generalized mixed linear model which were comparable with the results of the ANOVA used in the other two analyses.

In all three analyses, the predictors were test (pre-test/post-test, within subjects), group (control/experimental), and the interaction between these two predictors. Additionally, we controlled for autism, intellectual disability, RPM score, number of neurofeedback sessions (varied only in the experimental group), age, and gender. Autism and intellectual disability were coded as two separate binary variables and included as predictors in all statistical models. All analyses were performed in R (R Core Team, 2022).

## Results

There were no differences between the control and the experimental groups in gender (χ^2^[1] = 0.0059, *p* = 0.94), age (*t*[45.38] = 1.74, *p* = 0.09), or RPM score, (*t*[44.58] = 0.77, *p* = 0.44). As expected, the RPM score was related to diagnosis of intellectual disability (*t*[46.43] = − 7.71, *p* < 0.001). Mean score of participants diagnosed with autism was 48.4, while mean score of participants diagnosed with intellectual disability, including two participants with combined autism and intellectual disability diagnoses, was 31.24. Mean number of neurofeedback sessions in the experimental group was 20.17 (between 13 and 29, *SD* = 4.83). In the MCT, the mean proportion of false alarms was 1% in the pre-test and 2% in the post-test. Table 1 presents correlations and descriptive statistics of the main demographic and clinical traits and all dependent variables in the pre-test and post-test.

**Table 1:** Pearson correlations and descriptive statistics of main demographic and clinical traits and all dependent variables in pretest and posttest

	2	3	4	5	6	7	8	9	10	11	12
1. Male	− .11	− .36*	.43**	.28	.00	.23	.19	− .14	− .10	.22	.19
2. Age		.33*	− .28	− .18	.27	− .12	− .25	.06	− .11	− .19	− .24
3. Intellectual disability			− .83***	− .66***	− .09	− .33*	− .47***	− .08	.02	− .28*	− .23
4. Autism				.68***	.08	.41**	.43**	.05	.01	.32*	.30*
5. RPM					− .03	.53***	.57***	.25	.11	.64***	.62***
6. NFB Ses						.02	.08	.04	− .16	− .14	− .03
7. FST I							.75***	.26	.06	.41**	.52***
8. FST II								.14	.13	.45***	.54***
9. ALT I									.33 *	.18	.13
10. ALT II										.32*	.17
11. MCT I											.84***
12. MCT II											
Mean	17.68			36.49	9.88	2.69	2.81	18.84	21.53	10.08	8.04
SD	1.61			12.03	10.73	0.44	0.49	5.57	4.03	13.49	11.12
Range	[15, 22]			[12, 57]	[0, 29]	[1.08, 3.63]	[0.69, 3.95]	[5, 27]	[12, 27]	[0, 48]	[0, 46]

NFB Ses.—number of neurofeedback sessions; I, II—results in pre-test and post-test, respectively. In the case of gender, a point-biserial correlation was used

*p < .05, **p < .01, ***p < .001

There were main effects of both test (*M*_1_ = 2.69, *M*_2_ = 2.81, *F*[1,47] = 6.54, *p* = 0.014) and group (*M*_C_ = 2.78, *M*_E_ = 2.72, *F*[1,41] = 5.99, *p* = 0.019) on the combined performance (*d*’) in FST, but there was no interaction between these variables (*F*[1,47] = 0.07, *p* = 0.79). Moreover, there were effects of number of sessions (*B* = 0.042, *F*[1,41] = 6.76, *p* = 0.013) and RPM score (*B* = 0.015, *F*[1,41] = 5.64, *p* = 0.022, marginal *R*^2^ = 0.37, conditional *R*^2^ = 0.76). The complete set of ANOVA results is shown in Table 2. Because we observed that the number of neurofeedback sessions was a significant co-variate, we performed a follow-up analysis to determine whether the number of sessions was a significant predictor of the pre-test–post-test gain in the experimental condition by examining the interactive effect of test and number of trials on *d*’. The interaction between test and number of sessions in the experimental condition appeared to be nonsignificant (*F*[1,22] = 0.9, *p* = 0.35).

**Table 2:** Results of analysis of variance/deviance for all three tasks and complete set of predictors

Predictor	Task					
	FST		ALT		MCT	
	*F* (*df*)	*p*	*F* (*df*)	*p*	χ^2^ (*df*)	*p*
Test	6.54 (1,47)	.014*	10.79 (1,47)	.0019**	21.58 (1)	< .001***
Group	5.99 (1,41)	.019*	0.015 (1,41)	.9	2.41 (1)	.12
Test × Group	0.07 (1,47)	.79	0.97 (1,47)	.33	14.08 (1)	< .001***
Autism	0.32 (1,41)	.58	0.05 (1,41)	.82	1.04 (1)	.31
Intellectual disability	0.02 (1,41)	.89	0.079 (1,41)	.78	0.94 (1)	.33
RPM score	5.64 (1,41)	.022*	2.97 (1,41)	.092	29.79 (1)	< .001***
Number of sessions	6.76 (1,41)	.013*	0.004 (1,41)	.95	1.66 (1)	.2
Age	1.07 (1,41)	.3	0.0016 (1,41)	.97	0.34 (1)	.56
Gender	0.0084 (1,41)	.93	1.5 (1,41)	.23	2.28 (1)	.13

*p < .05, **p < .01, ***p < .001

There was a difference between the pre-test and post-test in the ALT score (*M*_1_ = 18.84, *M*_2_ = 21.53, *F*[1,47] = 10.79, *p* = 0.002). However, there was no effect of interaction between test and group (*F*[1,47] = 0.97, *p* = 0.33). Neither was there a main effect of group (*F*[1,41] = 0.015, *p* = 0.9) or any of the covariates (marginal *R*^2^ = 0.14, conditional *R*^2^ = 0.42). The complete set of ANOVA results is shown in Table 2.

**Fig. 3:**
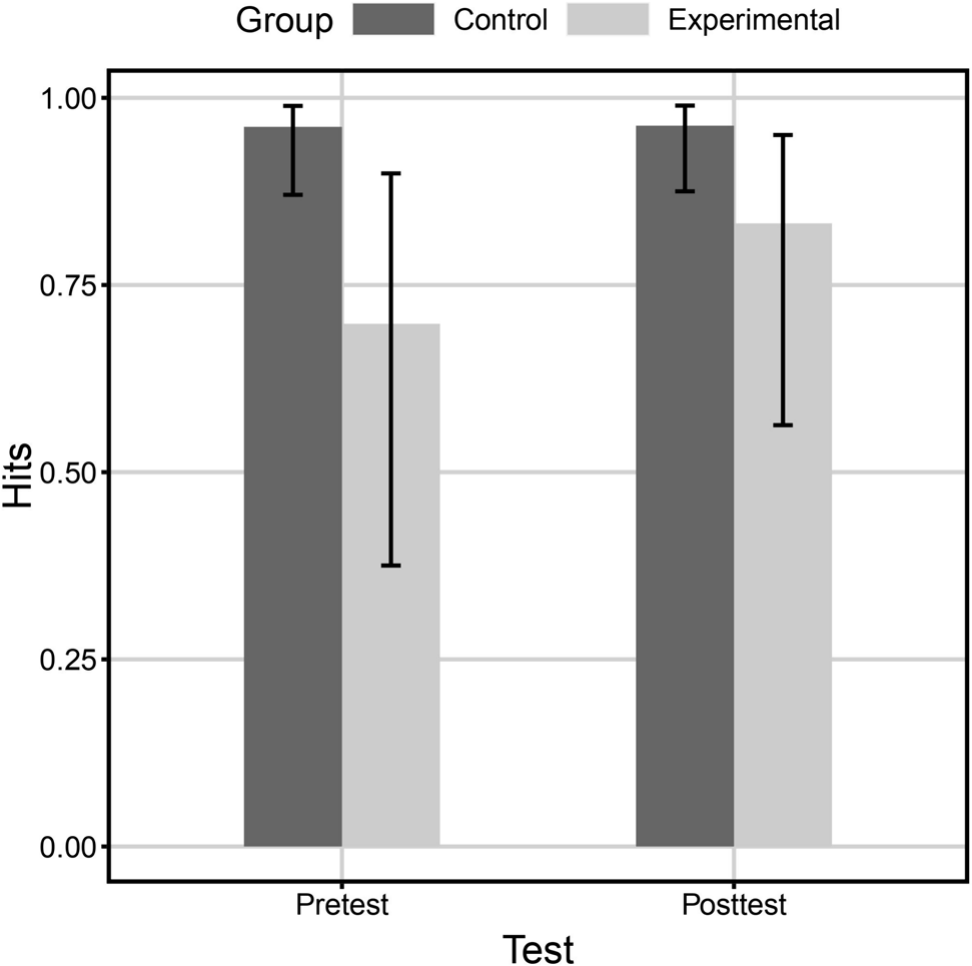
The effect of the interaction between test and group on the proportion of hits in MCT. The vertical lines mark 95% confidence intervals for estimation

In the MCT, participants performed better in the post-test than in the pre-test (*M*_1_ = 10.08, *M*_2_ = 8.04, χ^2^[1] = 21.58, *p* < 0.001) and there was no difference between groups (χ^2^[1] = 2.31, *p* = 0.12); however, the interaction between test and group influenced the number of hits (χ^2^[1] = 14.08, *p* < 0.001). Figure 3 presents the form of the interaction. Also, the RPM score appeared to be a significant positive predictor of performance (*B* = 0.015, χ^2^[1] = 29.79, *p* < 0.001, marginal *R*^2^ = 0.56, conditional *R*^2^ = 0.97). The complete set of results of the analysis of deviance is shown in Table 2.

## Discussion

In our study we aimed to determine whether school-based NFB training has a positive effect on the attention of people with autism or intellectual disabilities. We used behavioral test measures to assess selective attention, sustained attention in cognitively demanding situations, and sustained attention to simple stimuli. Our results indicate that one of the three tested aspects of attention was significantly improved after NFB training in the experimental group in comparison to controls. Specifically, we found that people in the experimental group obtained higher scores in the post-test than in the pre-test in sustained attention to simple stimuli, and no such improvement was observed in the control group. When it comes to selective attention, both groups’ performance improved in the post-test condition, and the control group performed significantly better than the experimental group. Improvement in selective attention in the experimental group was not predicted by the number of NFB sessions. Also, participants’ sustained attention performance improved in the post-test condition in the cognitively demanding situation, but this improvement was not related to NFB treatment.

Our results indicate that NFB treatment may improve the attentional functioning of people with autism and intellectual disability. However, as in previous studies which used behavioral test measures (Kouijzer et al. 2009a; 2010), we noted that the effects of NFB may be rather specific and related to basic cognitive functions and not to general attentional functioning. This improvement in sustained attention to simple stimuli stands in accordance with the results of studies on participants from the general population (Pamplona et al., 2020; Wang & Hsieh, 2013). Also, our study confirms that the skills of people with intellectual disability, a group that is relatively rarely subjected to NFB studies (Breteler et al., 2012; Hong & Lee, 2012; Surmeli & Ertem, 2007), may also be enhanced during NFB treatment. The results we obtained give further credence to the effectiveness of NFB therapy. However, our results also indicate that the effects of NFB treatment may be quite specific and should be further investigated in studies which involve larger samples and more rigorous methods of control (Thibault et al., 2017). Furthermore, the fact that the participants’ improved performance in attentional tasks could not be linked to the NFB training suggests that the results of studies on NFB efficacy that do not use control groups (e.g., Saleem & Habib, 2023 or Surmeli & Ertem, 2007) should not be treated as reliable evidence. One explanation for the increased performance in attentional tasks may concern the fact that students achieved better results simply because they were doing the tasks for the second time and could have benefitted from their previous experience with them. A more likely explanation concerns the fact that the students’ second performance in the attentional tasks was better due to their participation in various cognitively stimulating educational activities related to their school curriculum throughout the schoolyear. In our study design, we did not gather specific information regarding participation in different types of school activities.

As for the strengths of our study, it is important to note that we used objective test measures, which should give more accurate and credible information regarding the actual improvement in functioning than the third-party reports that are frequently used in studies on the efficacy of NFB. Also, we assessed the efficacy of NFB in a long-term perspective in a school setting, and the procedure we used may be easily implemented and repeated in other educational institutions. Our results provide modest evidence for the effects of NFB training on attention, but before implementing this treatment in any institution it is important to consider the related financial and organizational costs.

### Limitations and Future Directions

Our study has several limitations. First, although participants in the experimental and control groups had similar diagnoses and did not differ in their level of cognitive functioning, as measured by the RPM score, we did not pair students by diagnoses or individual test results. Also, several participants in our study were diagnosed with both autism and intellectual disability, therefore we could not separate the results for these two diagnoses in the analyses. Second, the sample in our study was relatively modest, although it is still larger than in most previous studies of people with autism or intellectual disability. The sample size in our study was related to the number of students who underwent NFB treatment at the institution where the research was conducted. Also, we did not control effects that participation in activities related to the school curriculum might have had on participants’ performance. It seems possible that stimulation provided in the educational setting during the year might have influenced the participants’ functioning. Finally, in our study we did not differentiate participants in regard to parameters that could potentially influence the results, such as their motivation or specific skills related to intellectual or executive functioning. Future studies should consider different psychological aspects of functioning that could potentially influence the efficacy of NFB.

An important point as to whether NFB therapy can be useful and worth the invested time for participants with autism and intellectual disabilities concerns the transfer of therapy effects to real-life situations. Based on the results of our study, we may only assume that the improvement in sustained attention that we observed should be beneficial to participants outside of NFB training sessions and that, for example, it could help them to achieve better performance in school tasks which require prolonged focus on material at hand. Future research should address the transfer effects of NFB therapy to real-life situations in people with autism and intellectual disabilities. Also, in this context, it is important to test students further in follow-up studies in order to assess whether their attentional skills change after the summer break from school, which in Poland lasts for two months. Such follow-up assessment should make it possible to obtain information on whether the effects of treatment on attention persist when no therapy is provided for a prolonged period of time.

## Conclusions

We assessed the efficacy of school-based NFB treatment in group of students diagnosed with autism and intellectual disabilities. The experimental group improved significantly in a task which measured sustained attention to simple stimuli. We did not observe effects of NFB treatment on selective attention or sustained attention in cognitively demanding situations. Further studies regarding NFB treatment in school settings should be conducted in which different contextual, motivational and cognitive variables potentially related to the efficacy of treatment will be considered.

## Data Availability

The data supporting the findings of this study is placed in public repository (10.17605/OSF.IO/NF83W).
